# Editorial: Reviews in cancer genetics

**DOI:** 10.3389/fonc.2023.1141161

**Published:** 2023-02-23

**Authors:** Zhanjun Guo, Patrícia S de Araujo-Souza, Shmuel Jaffe Cohen

**Affiliations:** ^1^ Department of Rheumatology and Immunology, The Fourth Hospital of Hebei Medical University, Shijiazhuang, China; ^2^ Laboratory of Immunogenetics and Histocompatibility, Universidade Federal do Paraná, Curitiba, Brazil; ^3^ Laboratory of Immuno-HPB and Transplant Surgery, Division of Surgery, Tel Aviv Medical Center, affiliated to the Sackler Faculty of Medicine, Tel Aviv University, Tel Aviv, Israel

**Keywords:** cancer, genes, oncogenes, genetic variants, reviews

Li et al. conduced a systematic review and meta-analysis to evaluate the association between Matrix Metalloproteinase 9 (MMP-9) expression and endometrial cancer (EC) risk, clinical features, and prognosis. MMP-9 is involved in many biological processes such as proteolytic degradation of extracellular matrix (ECM), cleavage of cell surface proteins, and alteration of cell-cell or cell-ECM interactions (1). Endometrial carcinoma can invade the basement membrane and myometrium through gelatinase, penetrating the lymphatic vascular lumen and spreading (2). MMP-9 gene was located at chromosome 20q13.12, which encoded Gelatinase B. Gelatinase B degraded gelatin, collagen, and elastin through proteolytic cleavage to regulate extracellular matrix (ECM) remodeling (3). A total of 28 eligible studies were acquired. MMP-9 overexpression was found to be significantly associated with the risk, tumor grade, FIGO stage, lymph node metastasis, and myometrium invasion of EC. In addition, the overall results showed that MMP-9 overexpression predicted a worse prognosis of EC (OR = 1.82, 95% CI = 1.01-2.62, P < 0.05).

Ding et al. reviewed the formation and regulatory mechanism of circRNAs, their biological function, and their relationship with gastric cancer. Differential expressions of sixty reviewed circRNAs were found to be upregulated in 35 cases of gastric cancer, mostly analyzed by RT-PCR. Studies have found that some circRNAs may act as oncogenes to promote, or as tumor suppressors to inhibit the occurrence and development of gastric cancer. Diagnostic markers, therapeutic targets, and prognostic markers for gastric cancer were deeply reviewed.

Montella et al. began their review article on genetic alterations in Glioblastoma (GBM) with the question “Is Genomics the Right Path?” In this review, the authors examined the most relevant molecular drivers of GBM from an interesting point of view, emphasizing the frustrating gap between translational research and its success in clinical applications. The article reviewed different up-to-date targeting strategies and clinical trial successes of important genetic alterations found in GBM: the telomerase reverse transcriptase gene, receptor tyrosine kinases, epidermal growth factor signaling, RAS, as well as the downstream cascade of kinases (mitogen-activated protein kinase (MAPK) and extracellular-regulated kinase (ERK)), neurofibromatosis 1 gene, mesenchymal–epithelial transition, fibroblast growth factor receptor, and the neurotrophic tyrosine receptor kinase family.

Zhang et al. reviewed the current understanding of the inhibitor of the apoptosis protein-related-like protein 2 (ILP-2) structure and function, as well as its potential application in cancer therapy. *BIRC8* (ILP-2 coding gene) is overexpressed in several tumors and can contribute to tumor immune evasion due to its role in apoptosis inhibition. The authors discussed its role as a biomarker for early tumor detection and the possibility of targeting ILP-2, associated with other cancer treatments, which can expand the options for cancer patients.

Yang et al. systematically searched for publications before 25 August 2021 to analyze the connections between CHRNA SNPs and lung cancer (LC) or chronic obstructive pulmonary disease (COPD). A total of 70,960 cases and 124,838 controls from 29 publications were identified for meta-analysis based on at least three data sources. Eight CHRNA SNPs (rs1051730, rs12914385, rs578776, rs6495309, rs8042374, and rs938682 in CHRNA3, as well as rs16969968 and rs588765 in CHRNA5) were identified to be associated with LC or COPD risk. Of them, rs1051730, rs6495309, and rs16969968 were significantly associated with COPD susceptibility, whereas rs1051730, rs578776, rs6495309, rs938682, rs16969968, and rs588765 were significantly associated with LC risk. By constructing functional annotations with the ENCODE project and other public databases, the authors found that these six SNPs may locate in several putative regulatory areas. In brief, this study found the variants of CHRNA genes associated with the risk of LC or COPD.

## Author contributions

All authors made direct and intellectual contributions to this article and provided their approval for the publication.
